# A checklist designed to aid consistency and reproducibility of GRADE assessments: development and pilot validation

**DOI:** 10.1186/2046-4053-3-82

**Published:** 2014-07-24

**Authors:** Nick Meader, Kristel King, Alexis Llewellyn, Gill Norman, Jennifer Brown, Mark Rodgers, Thirimon Moe-Byrne, Julian PT Higgins, Amanda Sowden, Gavin Stewart

**Affiliations:** 1 Centre for Reviews and Dissemination, University of York, York YO10 5DD, UK; 2 School of Social and Community Medicine, University of Bristol, Bristol BS8 2PS, UK; 3 School of Agriculture, Food and Rural Development, Newcastle University, Newcastle upon Tyne NE1 7RU, UK

**Keywords:** GRADE, Checklist, Quality assessment

## Abstract

**Background:**

The grading of recommendation, assessment, development and evaluation (GRADE) approach is widely implemented in health technology assessment and guideline development organisations throughout the world. GRADE provides a transparent approach to reaching judgements about the quality of evidence on the effects of a health care intervention, but is complex and therefore challenging to apply in a consistent manner.

**Methods:**

We developed a checklist to guide the researcher to extract the data required to make a GRADE assessment. We applied the checklist to 29 meta-analyses of randomised controlled trials on the effectiveness of health care interventions. Two reviewers used the checklist for each paper and used these data to rate the quality of evidence for a particular outcome.

**Results:**

For most (70%) checklist items, there was good agreement between reviewers. The main problems were for items relating to indirectness where considerable judgement is required.

**Conclusions:**

There was consistent agreement between reviewers on most items in the checklist. The use of this checklist may be an aid to improving the consistency and reproducibility of GRADE assessments, particularly for inexperienced users or in rapid reviews without the resources to conduct assessments by two researchers independently.

## Background

The grading of recommendation, assessment, development and evaluation (GRADE) approach provides guidance on rating the quality of research evidence in health care [1]. This approach has been widely implemented by organisations such as the World Health Organization, Cochrane Collaboration, Agency for Healthcare Research and Quality (USA) and National Institute of Health and Care Excellence (UK).

The GRADE approach is comprehensively described in an online manual (freely available for download with the GRADEpro software at http://tech.cochrane.org/revman/gradepro). This is summarised in a series of papers published in the *BMJ* in 2008 [2] and is also explained in a more detailed collection of papers in the *Journal of Clinical Epidemiology* (*JCE*) beginning in 2011 [1,3-15]. The GRADE approach is used to assess the quality of evidence for a specific outcome across studies. It applies most directly to a meta-analysis undertaken in the context of a systematic review but can be applied also to individual studies or non-quantitative syntheses when meta-analyses are not available. Evidence from randomised controlled trials (RCTs) begins as high-quality evidence but can be downgraded according to five factors: risk of bias, inconsistency, indirectness, imprecision and publication bias. Evidence from non-randomised studies begins as low-quality evidence, but their rating can be upgraded (provided no other limitations have been identified according to the five factors). Upgrading occurs for three primary reasons: large magnitude of effect, evidence of a dose-response effect and all plausible confounding taken into account. After the process of downgrading or upgrading, the quality of the evidence for each outcome ends up rated as high, moderate, low or very low. For the purposes of this paper, we will focus on the application of GRADE to meta-analyses of RCTs.

A major advantage of GRADE is that it leads to more transparent judgements about the quality of evidence [16]. The five key specific factors that may lead to downgrading the quality of evidence (and the need to state reasons for each downgrade) help to provide a clear rationale for such judgements. In addition, there is detailed written guidance from the GRADE working group for making these judgements. A further advantage of the broader GRADE framework is its ability to indicate the strength of recommendations based on the evidence.

However, a challenge to this extensive guidance is the complexity of the approach. There are substantial conceptual challenges when an organisation moves from previous methods of assessing overall study quality to implementing GRADE [16]. For example, it may be difficult for a researcher (particularly if they have limited experience of systematic reviews and/or GRADE) to remember and apply this guidance in a consistent manner (both within assessments as an individual, and between researchers in the same team) in the often time-pressured environment of conducting systematic reviews and guideline development.

Two recent empirical studies have examined the consistency of GRADE assessments. Hartling et al. [17] found poor-to-moderate agreement (based on kappa values) between researchers experienced in systematic review methods on rating quality of evidence. The GRADE working group [18] recently conducted an evaluation of consistency which was stratified by level of experience of the raters (members of the GRADE working group, students on the health research methodology programme at McMaster University). Inter-rater agreement was moderate when comparing individual ratings conducted independently by two researchers for both experienced members of the GRADE working group and for members of the student group. Inter-rater agreement was higher for members of the GRADE working group, when ratings were based on two individuals working independently who then reached a consensus judgement, then compared this judgement with another pair using the same consensus methods. Both studies suggest that GRADE ratings may not result in sufficient inter-rater agreement if conducted only by one assessor. Two experienced reviewers conducting a GRADE assessment in duplicate appears to result in acceptable agreement. However, this is less clear for inexperienced users of GRADE [18]. Therefore, further resources may improve consistency in conducting GRADE assessments by reviewers with less experience or for those with insufficient time and resources to conduct assessments by two reviewers independently.

We developed a checklist for evaluating meta-analyses of RCTs for the purpose of informing a GRADE assessment. The checklist covers the main determinants for each of the five factors (risk of bias, inconsistency, indirectness, imprecision, publication bias) that can lead to a downgrading of quality in the GRADE system. In this paper, we describe the development of the checklist and report on agreement between independent raters on each of the constituent items.

## Methods

### Developing the checklist

**Figure 1:**
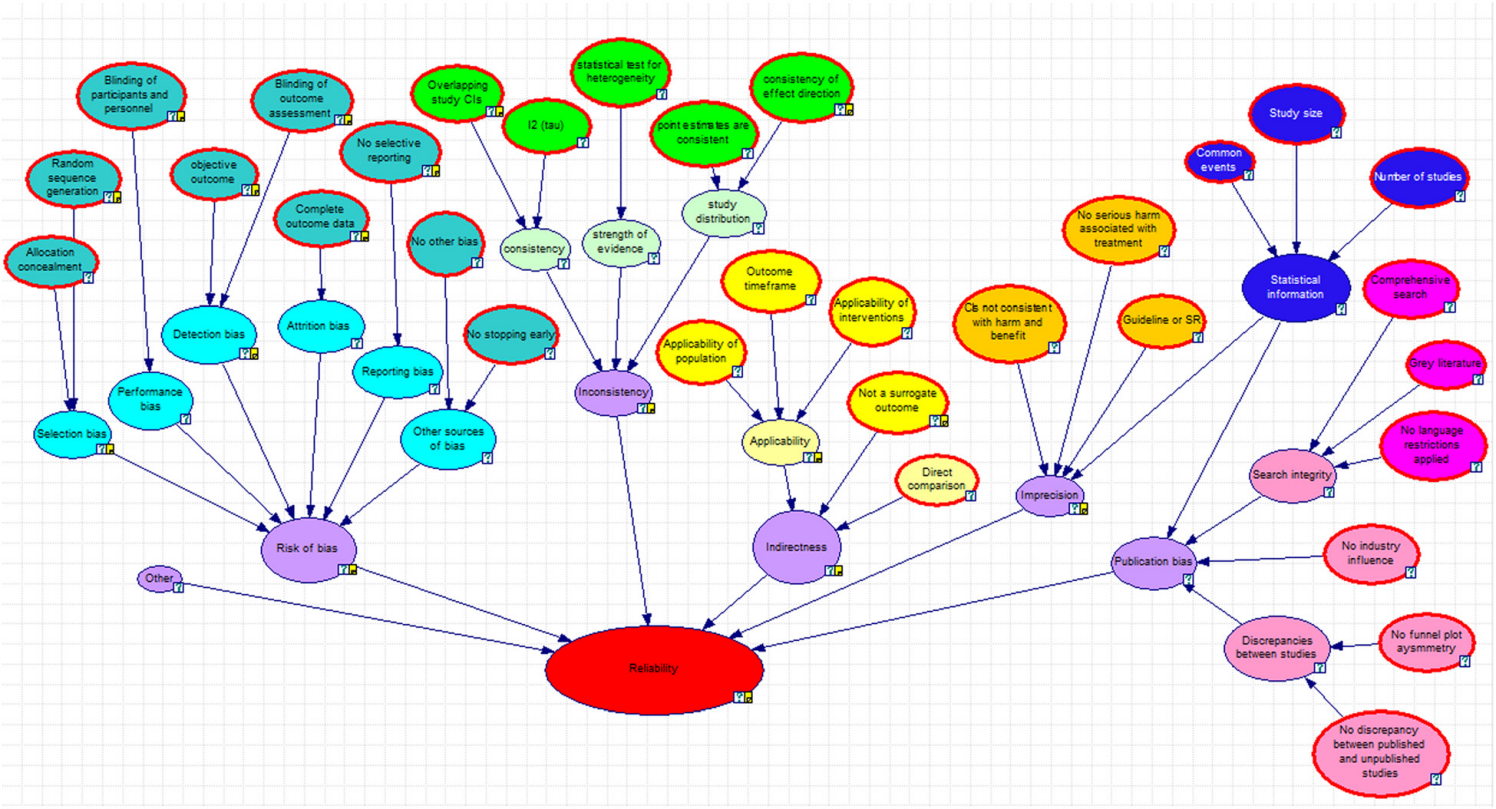
**Logic model for developing the checklist based on GRADE criteria.** A logic model illustrating how the checklist items relate to the risk of bias, inconsistency, indirectness, imprecision and publication bias domains in GRADE.

Two authors (GS, NM) drew a logic model which aimed to represent the GRADE assessment process (see Figure 1). The logic model comprises nodes for the various characteristics and properties of the evidence, connected with arrows to indicate how these attributes impact on each other. Population of the model was based on articles written by the GRADE working group with particular emphasis on the most recent series of articles in the *JCE*[1,3-15].

From this logic model, we developed a checklist of questions to extract the data required for conducting a GRADE assessment (see Figure 1). The full checklist appears in Additional file 1. Where possible, all questions were derived as directly as possible from literature produced by the GRADE working group. However, where specific guidance was lacking, some of the questions included our value judgements (e.g. what constitutes a low, medium or high value of *I*^2^; or the extent to which confidence intervals overlap; or what constitutes a high dropout rate).

Questions about study limitations (risk of bias) were based on items in the Cochrane risk of bias tool [19] as suggested by the GRADE working group [6]. They are to be answered in relation to the majority of the aggregated evidence in the meta-analysis rather than to individual studies. Questions about inconsistency were based primarily on visual assessment of forest plots and the statistical quantification of heterogeneity based on *I*^2^ and *Q* statistics. This includes assessments of subgroups (particularly those defined *a priori*) that appear to explain the inconsistency.

Indirectness items included questions on the applicability of the population, intervention, comparator and outcome (whether a surrogate or not, and whether follow-up time was sufficient) based on the majority of the aggregated evidence in the meta-analysis. The checklist does not specifically address reviews using network meta-analysis (as there is not currently any published guidance on this). However, the checklist did consider indirectness in terms of informal indirect comparisons made between interventions based on pairwise meta-analysis.

Imprecision was addressed through items relating to the width of the confidence interval and sample size. When judging the width of the confidence interval, GRADE recommends that reviewers use a clinical decision threshold to assess whether the imprecision is clinically meaningful [8]. We did not explicitly build this into the checklist, although the initial question about whether the estimate is consistent with benefit and harm might be interpreted in relation to a minimally important difference.

Questions about publication bias addressed comprehensiveness of the search strategy, whether included studies had industry influence and funnel plot asymmetry and whether there was evidence of discrepancies between published and unpublished trials.

We focused specifically on meta-analyses of RCTs. Therefore, we did not include factors to upgrade the evidence (magnitude of effect, dose-response relationship, adjusting for known confounding) as these are recommended for use only in the context of systematic reviews of non-randomised studies where downgrading was not required for any of the five potential factors.

### Initial piloting of the checklist

We conducted two phases of initial piloting of these questions. First, the wider project team applied the draft checklist independently to five systematic reviews and then met to discuss their assessments and any difficulties they experienced. Second, a set of 15 systematic reviews was assessed by two reviewers independently to identify any further potential difficulties. Both phases led to some refining of the instrument. One of the challenges identified by the team in the second phase was that the response ‘yes’ referred to something positive (e.g. was the sequence generation randomised?) in some items and negative in others (e.g. was there selective reporting?). Several reviewers suggested it would be more straightforward to use the checklist if all questions were worded so that ‘yes’ always referred to a positive feature of the quality of evidence and ‘no’ to something negative (where yes and no responses were required). Therefore, we included this modification in the main validation of the checklist.

### External validation of the checklist

Following the development and initial piloting of the checklist, we conducted a more formal evaluation of its inter-rater agreement. We examined 29 systematic reviews containing a meta-analysis of RCTs included in the Database of Abstracts of Reviews of Effects (DARE) [20-48] (Table 1). One author (NM) selected the reviews to ensure variety both in terms of reporting and reliability based on an informal assessment of the systematic reviews. Papers were selected to ensure a diverse range of reviews from a variety of disease areas including cardiology, diabetes, chronic obstructive pulmonary disease, neurological conditions (dementia, stroke, Parkinson's disease), oncology (non-small cell lung cancer, ovarian cancer, metastatic colorectal cancer) and prevention of pancreatitis, osteoarthritis, restless legs syndrome and sciatica. In addition, reviews on substance misuse (opioid detoxification, psychostimulant dependence), mental health (depression, anxiety, obsessive compulsive disorder, attention deficit hyperactivity disorder) and HIV prevention were also included. Pharmacological and non-pharmacological interventions were included.

**Table 1:** Brief summary of systematic reviews included in pilot evaluation

**Author**	**Title**	**Intervention type**	**Topic area**
AHRQ [20]	Comparative effectiveness review 86: treatment for restless legs syndrome	Pharmacological	Neurology
Amato et al. [21]	Methadone at tapered doses for the management of opioid withdrawal	Pharmacological	Tobacco, drugs and alcohol
Andrews et al. [22]	Interventions to influence consulting and antibiotic use for acute respiratory tract infections in children: a systematic review and meta-analysis	Psychosocial	Respiratory infections
Andrews et al. [23]	Computer therapy for the anxiety and depressive disorders is effective, acceptable and practical health care: a meta-analysis	Computer delivered psychological	Mental health
Birks [24]	Cholinesterase inhibitors for Alzheimer's disease	Pharmacological	Neurology
Cape et al. [25]	Brief psychological therapies for anxiety and depression in primary care: meta-analysis and meta-regression	Psychological	Mental health
Chen et al. [26]	Gefitinib or erlotinib as maintenance therapy in patients with advanced stage non-small cell lung cancer	Pharmacological	Cancer
Cipriani et al. [27]	Escitalopram versus other antidepressive agents for depression	Pharmacological	Mental health
Eaton et al. [28]	Meta-analysis of single-session behavioural interventions to prevent sexually transmitted infections: implications for bundling prevention packages	Psychosocial	Infectious disease
Eggington et al. [29]	Care management for type 2 diabetes in the United States: a systematic review and meta-analysis	Service	Endocrine and metabolic
Eyding et al. [30]	Reboxetine for acute treatment of major depression: systematic review and meta-analysis of published and unpublished placebo and selective serotonin reuptake inhibitor controlled trials	Pharmacological	Mental health
Gould et al. [31]	Efficacy of cognitive behavioural therapy for anxiety disorders in older people: a meta-analysis and meta-regression of randomized controlled trials	Psychological	Mental health
Harkness et al. [32]	Identifying psychosocial interventions that improve both physical and mental health in patients with diabetes	Psychosocial	Mental health and endocrine and metabolic
Huedo-Medina et al. [33]	Efficacy of HIV prevention interventions in Latin American and Caribbean nations 1995-2008: a meta-analysis	Psychosocial	Infectious disease
Johnston et al. [34]	Probiotics for the prevention of *Clostridium difficile*-associated diarrhea: a systematic review and meta-analysis	Probiotics	Infectious disease
Makani et al. [35]	Efficacy and safety of dual blockade of the rennin-angiotensin system: meta-analysis of randomised trials	Pharmacological	Heart and circulation
Minns Lowe et al. [36]	Effectiveness of physiotherapy exercise after knee arthroplasty for osteoarthritis: systematic review and meta-analysis of randomised controlled trials	Physical	Rheumatology
Nelson et al. [37]	A systematic review and meta-analysis of placebo-controlled antidepressant studies in people with depression and dementia	Pharmacological	Mental health and neurology
Perez-Mana et al. [38]	Efficacy of indirect dopamine agonists for psychostimulant dependence: a systematic review and meta-analysis of randomised controlled trials	Pharmacological	Tobacco, drugs and alcohol
Pinto et al. [39]	Epidural corticosteroid injections in the management of sciatica: a systematic review and meta-analysis	Pharmacological	Rheumatology
Preiss et al. [40]	Lipid-modifying therapies and risk of pancreatitis: a meta-analysis	Pharmacological	Heart and circulation
Rayner et al. [41]	Antidepressants for depression in physically ill people	Pharmacological	Mental health
Skapinakis et al. [42]	Efficacy and acceptability of selective serotonin reuptake inhibitors for the treatment of depression in Parkinson's disease: a systematic review and meta-analysis of randomized controlled trials	Pharmacological	Mental health and neurology
Soomro et al. [43]	Selective serotonin re-uptake inhibitors (SSRIs) versus placebo for obsessive compulsive disorder	Pharmacological	Mental health
Wang et al. [44]	Effect of long-acting beta-agonists on the frequency of COPD exacerbations: a meta-analysis.	Pharmacological	Respiratory infections
Wardlaw et al. [45]	Thrombolysis for acute ischaemic stroke	Pharmacological	Neurology
Ye et al. [46]	Bevacizumab in the treatment of ovarian cancer: a meta-analysis from four phase III randomized controlled trials	Pharmacological	Cancer
Zhang et al. [47]	Capecitabine plus oxaliplatin compared with 5-fluorouracil plus oxaliplatin in metastatic colorectal cancer: meta-analysis of randomized controlled trials	Pharmacological	Cancer
Zhong [48]	Chemotherapy plus best supportive care versus best supportive care in patients with non-small cell lung cancer: a meta-analysis of randomized controlled trials	Pharmacological	Cancer

The checklist was used by two reviewers independently for all selected reviews. For each review, one author (NM) developed a review question in terms of the population, intervention, comparison and outcome (PICO) of interest. One critical outcome (the primary outcome) was selected for assessment for each review. GRADE recommends a maximum of nine outcomes, but we limited it to one outcome for the purposes of this study to enable reviewers to assess a wider sample of reviews.

Reviewers based their assessments on the information reported in the paper relating to the systematic review and did not seek to obtain information from the original reports of included RCTs (other than for evidence of industry involvement which was rarely reported in systematic reviews and therefore original papers of the largest studies in the meta-analysis were checked).

We calculated a weighted kappa statistic with 95% confidence interval (CI) for each item of the checklist. We interpreted the coefficients according to the following guidelines: below chance was considered poor; 0.01 to 0.20 slight agreement; 0.21 to 0.40 fair agreement; 0.41 to 0.60 moderate agreement; 0.61 to 0.80 substantial agreement; and 0.81 to 1 almost perfect agreement [49]. Each reviewer recorded the time spent conducting an assessment, and from these data, we summarised these to estimate the likely resource implications of using this approach.

### Experience of reviewers

Seven reviewers from the Centre for Reviews and Dissemination (CRD) participated in the checklist assessments. CRD specialises in conducting, critically appraising and developing methods for evidence syntheses and systematic reviews. Years of systematic review experience among participants ranged from 2 to 10. Although six reviewers had no formal training or experience using GRADE, all were experienced in critical appraisal and validity assessment (of both primary studies and systematic reviews), for example, the Cochrane risk of bias tool and quality assessment according to criteria developed by CRD.

Two authors (GS, NM) provided informal GRADE training during the course of the study and provided guidance on completing the checklist.

## Results

### Judgements for each of the checklist items

Measures of agreement for all specific items in the checklist are given in Table 2. For most of the items designed to examine risk of bias, agreement was found to be either almost perfect or substantial. For one item, designed to measure attrition bias, agreement was moderate. For items concerning no other bias and selective reporting, level of agreement was relatively low.

**Table 2:** Agreement for all checklist items

**Item**	**Kappa (95% CI)**	**Magnitude of agreement**
Risk of bias		
Was random sequence generation used (i.e. no potential for selection bias)?	0.89 (0.69 to 1)	Almost perfect
Was allocation concealment used (i.e. no potential for selection bias)?	0.69 (0.29 to 1)	Substantial
Was there blinding of participants and personnel (i.e. no potential for performance bias)?	0.71 (0.41 to 1)	Substantial
Was there blinding of outcome assessment (i.e. no potential for detection bias)?	0.98 (0.67 to 1)	Almost perfect
Was an objective outcome used?	1	Almost perfect
Were more than (80%)^a^ of participants enrolled in trials included in the analysis? (i.e. no potential attrition bias)	0.44 (0.07 to 0.81)	Moderate
Were data reported consistently for the outcome of interest (i.e. no potential selective reporting)? (no potential reporting bias)	0.25 (0 to 0.61)	Fair
No other biases reported? (no potential of other bias)	0.20 (0 to 0.62)	Slight
Did the trials end as scheduled (i.e. not stopped early)?	1	Almost perfect
Inconsistency		
Point estimates did not vary widely? (i.e. no clinical meaningful inconsistency)	0.65 (0.37 to 0.93)	Substantial
To what extent do confidence intervals overlap?	0.50 (0.17 to 0.77)	Moderate
Was the direction of effect consistent?	1	Almost perfect
What was the magnitude of statistical heterogeneity (as measured by *I*^2^)?	1	Almost perfect
Was the test for heterogeneity statistically significant (*p* < 0.1)?	1	Almost perfect
Indirectness		
Were the populations in included studies applicable to the target population?	Below chance	Poor
Were the interventions in included studies applicable to target intervention?	Below chance	Poor
Was the included outcome not a surrogate outcome?	1	Almost perfect
Was the outcome timeframe sufficient?	0.47 (0 to 1)	Moderate
Were the conclusions based on direct comparisons?	1	Almost perfect
Imprecision		
Was the confidence interval for the pooled estimate not consistent with benefit and harm?	1	Almost perfect
What was the magnitude of the median sample size?	1	Almost perfect
What was the magnitude of the number of included studies?	1	Almost perfect
Was the outcome a common event? (e.g. occurs more than 1/100)^a^	1	Almost perfect
Was there no evidence of serious harm associated with treatment?	0.89 (0.67 to 1)	Almost perfect
Publication bias		
Did the authors conduct a comprehensive search?	0.65 (0 to 1)	Substantial
Did the authors search for grey literature?	0.26 (0 to 0.67)	Fair
Authors did not apply restrictions to study selection on the basis of language?	0.74 (0.45 to 1)	Substantial
There was no industry influence on studies included in the review?	0.71 (0.45 to 0.98)	Substantial
There was no evidence of funnel plot asymmetry?	0.62 (0.35 to 0.89)	Substantial
There was no discrepancy in findings between published and unpublished trials?	1	Almost perfect

^a^These thresholds can be replaced with different ones based on the context of the particular review.

For all items on imprecision, there was almost perfect agreement which reflects the fact that less judgement is needed for these items, and therefore, a high level of agreement would be expected.

Four of the five items on inconsistency had almost perfect or substantial agreement. The item on the extent of overlap of confidence intervals was associated with moderate agreement.

Two of the items on indirectness (applicability of population and applicability of intervention) did not perform well, and the consistency was below chance. For two of the items (use of surrogate outcome and direct comparisons), agreement was almost perfect, and for one item moderate (sufficiency of follow-up time for outcome).

For five out of six items on publication bias, there was substantial or almost perfect agreement. Only fair agreement was found on the item for searching grey literature.

### Time required for conducting assessments

The median time to conduct the assessment was 30 min (range = 15–90 min). Informal assessment suggested that years of experience of reviewers and complexity of the review appeared to be the main factors impacting on variability of time taken to complete assessment.

Reviewers' feedback suggested that it was relatively straightforward to use the checklist despite almost all reviewers having no prior formal training or experience using GRADE.

## Discussion

Some of the main difficulties in applying the GRADE system are the complexity and cognitive demands of the approach and the potential lack of reproducibility of the judgements made. Our proposed checklist helps researchers to identify and extract the information needed for a GRADE assessment in a repeatable manner. In addition, it increases transparency because the items used to make the assessment are clearly identified.

For most checklist items (70%), there was substantial agreement or better, suggesting that most of the data required for a GRADE assessment can be extracted in a repeatable manner.

However, some problems were identified for checklist items relating to indirectness. There was a lack of agreement for most of these items, although there was good agreement on surrogate outcomes. This reflects GRADE guidance which acknowledges that considerable judgement is needed when considering the applicability with regard to populations and interventions [10]. In addition, it is likely that given the wide scope of the reviews being quality assessed, there may have been a lack of clarity about the target population and intervention. As assessments were conducted independently, it was not possible to discuss how to apply criteria for assessing indirectness (or consult with advisors with expertise in the subject area), which is common practice when working in a team of reviewers.

A potential limitation of this study is that we conducted the GRADE assessments on published reviews, therefore basing our judgement on the data reported in these reviews rather than as part of the process of conducting a systematic review. It is possible agreement would either increase or decrease if we used the latter approach. However, our approach reflects the reality of guideline development and of conducting overviews of reviews where evidence from existing systematic reviews will often be utilised to inform conclusions and/or recommendations. It is also consistent with the methods used in a previous evaluation of the inter-rater agreement for GRADE assessments [18].

A further potential limitation is that the systematic reviews we assessed were not randomly selected. However, for the purposes of this study, we were not aiming to provide a random sample of all systematic reviews. Instead, we wanted to ensure that reviews from a variety of disease areas and using different types of interventions were examined during the assessment of the performance of the checklist.

The checklist only includes criteria relating to systematic reviews that include meta-analyses of RCTs. Of course, the GRADE approach can be applied to non-randomised studies and also synthesis techniques other than meta-analysis. Further work is needed to adapt and validate this checklist for such reviews. A further possible limitation was that effort to make all ‘yes’ responses to questions reflect low bias may have resulted in awkward wording (e.g. use of double negatives) for some items. This may have reduced the user-friendliness of the checklist and potentially reduced inter-rater agreement.

While aiming to reduce some of the complexity of conducting GRADE assessments, it is important to note that reviewers still need to develop clearly defined review questions (including the selection of critical or important outcomes) before completing the checklist. In addition, using the checklist still requires the reviewer to make important value judgements. For example, as noted above, considerable judgement is needed to assess the applicability of populations, interventions and comparisons for a particular review question. Similarly, reviewers will need to develop thresholds to assess, for example, whether the width of the confidence interval for a pooled estimate (for questions on imprecision) and variability in study estimates (for questions on inconsistency) included in the meta-analysis constitute clinically meaningful threats to validity.

In addition, the issue of improving repeatability of GRADE assessments that we address in this paper is one among many challenges in the use of GRADE. One of these other challenges is that of how to decide whether to downgrade by none, one or two levels for issues identified in one of the five GRADE domains. Considerable guidance is available from GRADE on how these different factors should be weighted, and scenarios provided where a combination of factors should likely lead to a downgrade by one or two levels. Our checklist provides added structure for the information to inform this decision but does not necessarily highlight when there is sufficient evidence to justify any particular degree of downgrading.

To this end, we have developed a semi-automated quality assessment tool (SAQAT) which is a Bayesian network model that uses responses to the checklist questions to produce GRADE assessments. Although semi-automated approaches have not been widely used in critical appraisal of systematic reviews, they may offer an alternative for reviewers who struggle with the complexity of GRADE. Manuscripts about the SAQAT and its performance in practice are currently in development.

## Conclusions

In conclusion, experienced systematic reviewers but with little or no experience of conducting GRADE assessments appear to be able to answer our checklist of questions in a broadly consistent and reproducible manner when assessing the quality of evidence for meta-analyses of RCTs. Further work is needed to improve agreement on judgements relating to applicability of interventions and populations (as factors to consider within indirectness).

Use of our checklist is most likely to benefit those with limited experience of using GRADE. However, given the complexity of GRADE, we think the checklist may act as a helpful reminder for experienced users of the key factors to consider for each of the five GRADE domains (risk of bias, imprecision, inconsistency, indirectness and publication bias). Our checklist may offer improvements in efficiency and time and therefore may be beneficial when used in the context of a rapid review. For example, we found that inexperienced users of GRADE were able to complete an assessment in a median of 30 min.

Next steps include the need to pilot the use of this checklist when conducting systematic reviews and/or during guideline development and assess whether this results in more consistent judgements when conducting a GRADE assessment. It would also be important to assess further the utility of using the checklist for different review questions and disease areas, and the extent to which it might need to be adapted. We particularly encourage researchers and health technology assessment organisations currently using or considering using GRADE to pilot our tool and provide us with feedback to inform further development.

## Abbreviations

BMJ: British Medical Journal; CI: Confidence interval; CRD: Centre for Reviews and Dissemination; GRADE: Grading of recommendation, assessment, development and evaluation; RCT: Randomised controlled trial; JCE: Journal of Clinical Epidemiology.

## Competing interests

The authors declare that they have no competing interests.

## Authors' contributions

NM contributed to the development of checklist, designed the study, conducted data analysis and drafted the manuscript. KK, AL, GN, JB, TMB and MR contributed to the development of checklist, used the checklist on included systematic reviews and critically revised the manuscript. GS, AS and JPTH contributed to the design of the study and interpretation of data, and critically revised the manuscript. All authors read and approved the final manuscript.

## Supplementary Material

Additional file 1 **Checklist for the quality assessment tool.** The checklist covers the main determinants for each of the five factors: risk of bias, inconsistency, indirectness, imprecision and publication bias. Click here for file
