# Supplementary material for: A checklist designed to aid consistency and reproducibility of GRADE assessments: development and pilot validation
Source: Syst Rev. 2014 Jul 24;3:82. doi: 10.1186/2046-4053-3-82 (PMC4124503; doi:10.1186/2046-4053-3-82)
Supplement: Additional file 1 — Checklist for the quality assessment tool. The checklist covers the main determinants for each of the five factors: risk of bias, inconsistency, indirectness, imprecision and publication bias. [file 2046-4053-3-82-S1.doc]

**Checklist for the Quality Assessment Tool**

**Study limitations (Risk of Bias)**

1) Was random sequence generation used (i.e. no potential for selection bias)?

- Yes
- No
- Unclear

2) Was allocation concealment used (i.e. no potential for selection bias)?

- Yes
- No
- Unclear

3) Was there blinding of participants and personnel (i.e. no potential for performance bias)?

- Yes
- No
- Unclear

4) Was there blinding of outcome assessment (i.e. no potential for detection bias)?

- Yes
- No
- Unclear

5) Was an objective outcome used?

- Yes
- No

6) Were more than 80%[[1]](#footnote-2) of participants enrolled in trials included in the analysis (i.e. no potential reporting bias)?

- Yes
- No
- Unclear

7) Were data reported consistently for the outcome of interest (i.e., no potential selective reporting)?

- Yes
- No
- Unclear

8) No other biases reported? (i.e. no potential of other bias)

- Yes
- No

9) Did the trials end as scheduled (i.e not stopped early)?

- Yes
- No

**Inconsistency[[2]](#footnote-3)**

1) Point estimates did not vary widely?

- Yes
- No

2) To what extent did confidence intervals overlap?

- Substantial overlap

(all confidence intervals overlap at least one of the included studies point estimate)

- Some overlap

(confidence intervals overlap but not all overlap at least one point estimate)

- No overlap

(At least one outlier: where the confidence interval of some of the studies do not overlap with those of most included studies)

3) Was the direction of effect consistent?

- Yes
- No

4) What was the magnitude of statistical heterogeneity (as measured by I^2^)?

- Low (e.g. I^2^ <40%)
- Moderate (e.g. I^2^ 40-60%)
- High (e.g. I^2^ >60%)

5) Was the test for heterogeneity statistically significant (p<0.1)?

- Not statistically significant
- Statistically significant

**Indirectness**

1) Were the populations in included studies applicable to the decision context?

- Highly applicable
- Applicable
- Poorly applicable

2) Were the interventions in the included studies applicable to the decision context?

- Highly applicable
- Applicable
- Poorly applicable

3) Was the included outcome not a surrogate outcome?

- Yes
- No

4) Was the outcome timeframe sufficient?

- Sufficient
- Insufficient

5) Were the conclusions based on direct comparisons?

- Yes
- No

**Imprecision**

1) Was the confidence interval for the pooled estimate not consistent with benefit and harm?

- Yes
- No

2) What is the magnitude of the median sample size?

- High (e.g. 300 participants)
- Intermediate (e.g. 100-300 participants)
- Low (e.g. <100 participants)

3) What was the magnitude of the number of included studies?

- Large (e.g. >10 studies)
- Moderate (e.g. 5-10 studies)
- Small (e.g. <5 studies)

4) Was the outcome a common event (e.g. occurs more than 1/100)?

- Yes
- No
- Not applicable (i.e. not a dichotomous outcome)

*Further optional question for those engaged in guideline development[[3]](#footnote-4)*

5) Was there no evidence of serious harm associated with treatment?

- Yes
- No

**Publication Bias (other considerations)**

1) Did the authors conduct a comprehensive search?

- Yes
- No

2) Did the authors search for grey literature?

- Yes
- No

3) Authors did not apply restrictions to study selection on the basis of language?

- Yes
- No

4) There was no industry influence on studies included in the review?

- Yes
- No

5) There was no evidence of funnel plot asymmetry?

- Yes
- No
- Unclear

6) There was no discrepancy in findings between published and unpublished trials?

- Yes
- No
- Unclear

1. 80% drop out is given as an example here a different proportion can be used depending on the context of the systematic review area [↑](#footnote-ref-2)
2. Reviewers may choose to use estimates from a subgroup analysis which may explain the inconsistency but should be cautious that such a explanation of heterogeneity may be due to the play of chance [↑](#footnote-ref-3)
3. This reflects GRADE guidance that guideline developers may use a less stringent threshold for judging imprecision of an intervention’s benefits when there is no evidence of harm compared with when judging the benefits of an intervention where there is strong evidence of harm [↑](#footnote-ref-4)
